# Deep learning-based monocular placental pose estimation: towards collaborative robotics in fetoscopy

**DOI:** 10.1007/s11548-020-02166-3

**Published:** 2020-04-30

**Authors:** Mirza Awais Ahmad, Mouloud Ourak, Caspar Gruijthuijsen, Jan Deprest, Tom Vercauteren, Emmanuel Vander Poorten

**Affiliations:** 1 Department of Mechanical Engineering, KU Leuven, Leuven, Belgium; 2 Department of Development and Regeneration, KU Leuven, Leuven, Belgium; 3 Department of Obstetrics and Gynaecology, UZ Leuven, Leuven, Belgium; 4 School of Biomedical Engineering and Imaging Sciences, King’s College London, London, UK

**Keywords:** Deep learning, Convolutional neural networks, Robot assisted surgery, Shared control, Orientation estimation, Fetoscopy

## Abstract

**Purpose:**

Twin-to-twin transfusion syndrome (TTTS) is a placental defect occurring in monochorionic twin pregnancies. It is associated with high risks of fetal loss and perinatal death. Fetoscopic elective laser ablation (ELA) of placental anastomoses has been established as the most effective therapy for TTTS. Current tools and techniques face limitations in case of more complex ELA cases. Visualization of the entire placental surface and vascular equator; maintaining an adequate distance and a close to perpendicular angle between laser fiber and placental surface are central for the effectiveness of laser ablation and procedural success. Robot-assisted technology could address these challenges, offer enhanced dexterity and ultimately improve the safety and effectiveness of the therapeutic procedures.

**Methods:**

This work proposes a ‘minimal’ robotic TTTS approach whereby rather than deploying a massive and expensive robotic system, a compact instrument is ‘robotised’ and endowed with ‘robotic’ skills so that operators can quickly and efficiently use it. The work reports on automatic placental pose estimation in fetoscopic images. This estimator forms a key building block of a proposed shared-control approach for semi-autonomous fetoscopy. A convolutional neural network (CNN) is trained to predict the relative orientation of the placental surface from a single monocular fetoscope camera image. To overcome the absence of real-life ground-truth placenta pose data, similar to other works in literature (Handa et al. in: Proceedings of the IEEE conference on computer vision and pattern recognition, 2016; Gaidon et al. in: Proceedings of the IEEE conference on computer vision and pattern recognition, 2016; Vercauteren et al. in: Proceedings of the IEEE, 2019) the network is trained with data generated in a simulated environment and an in-silico phantom model. A limited set of coarsely manually labeled samples from real interventions are added to the training dataset to improve domain adaptation.

**Results:**

The trained network shows promising results on unseen samples from synthetic, phantom and in vivo patient data. The performance of the network for collaborative control purposes was evaluated in a virtual reality simulator in which the virtual flexible distal tip was autonomously controlled by the neural network.

**Conclusion:**

Improved alignment was established compared to manual operation for this setting, demonstrating the feasibility to incorporate a CNN-based estimator in a real-time shared control scheme for fetoscopic applications.

## Introduction

**Fig. 1:**
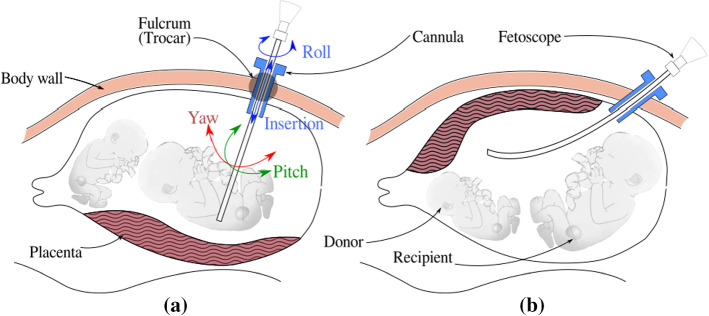
Laser coagulation for TTTS: **a** a straight rigid fetoscope is used in the case of posterior placenta **b** a curved rigid fetoscope for an anterior placenta

Twin-to-twin transfusion syndrome (TTTS) is a placental defect occurring in monozygotic twins that share a single placenta (monochorionic). This anomaly occurs in 10–15% of monochorionic twin pregnancies [15]. This placental defect causes disproportional blood transfusion among the twin fetuses through placental anastomoses. This leads to over-development of one of the twins (recipient) and under-development of the other twin (donor). This condition can be fatal for both of the fetuses and has near-100% mortality rate if not treated [3]. The suggested therapy is endoscopic laser ablation (ELA). In ELA, the surgeon enters the uterine cavity with an endoscope (fetoscope) having a working channel for passing a therapeutic laser. The anastomosing vessels on the shared placenta are identified visually and coagulated by laser ablation [19]. ELA for TTTS has become one of the most common fetoscopic operations today. The survival of at least one of the fetuses is in the range of 75–80% [15]. ELA requires only a small, typically 3mm diameter, incision in the uterine wall. Compared to hysterotomy, ELA decreases the procedure-induced morbidity significantly [26].

In the conventional approach, shown in Fig. 1, a rigid fetoscope is inserted through a cannula in a small incision into the uterine cavity. For effective vessel coagulation Deprest et al. [3] showed the importance of a clear visualization inside the amniotic sac, the possibility to view the complete inter-twin membrane for target vessel identification, and the possibility to position the laser fiber at an angle close to $90^{\circ }$ (thus perpendicular to the surface). The insertion site must be chosen carefully, in function of the position of the fetuses and of the location of the placenta to maximally comply with above factors. Especially for anterior placentas, when the placenta is attached to the front of the stomach (Fig. 1b) adequate insertion sites are scarce, possibly leading to sub-optimal coagulation. Classical rigid instruments offer little dexterity inside the womb. Surgeons, therefore, tend to exert overly large forces on the insertion point to gain maneuverability. However, such large forces could increase the risk for iatrogenic Preterm Premature Rupture of Membranes (iPPROM) [6] and should therefore be avoided.

In summary, shortcomings of the current instruments include: inability to achieve orthogonal orientation of the fetoscope and laser (more pronounced in the case of anterior placenta); the need to change scopes over different interventional stages; poor image stability (motion compensation schemes are absent); difficulty to prevent inadvertent ablation or tissue contact and feto-placental hemorrhage when operating in too close proximity to the surface [5]. In the literature, different types of scopes have been proposed to overcome these issues: a rigid endoscope with a rotating mirror at the distal tip [24]; a side-firing laser fiber or fiber deflecting mechanism [12]; flexible scopes that can bend both the laser fiber and the camera [9, 25, 27]. Rigid scopes cannot re-orient the camera and suffer from limited range of visibility. The flexible instruments in the literature provide more degrees of freedom (DoFs) but they can so far only accommodate one single tool at a time, either a camera, laser fiber or forceps. For TTTS both a camera, illumination *and* a laser fiber are needed. In our previous work an instrument with a rigid shaft and a steerable flexible distal tip was presented. The instrument has a chip-on-tip camera, a fiber-optic light source and a working channel for laser fiber. The design, usability and shared control approaches have been discussed in [1, 14]. Due to usability issues and steep learning curves, a shared control approach was proposed where the surgeon was in charge of the gross instrument motion and the distal tip moved autonomously. A sensing system would be needed for autonomous control, but as size limitations are severe, adding extra sensors is no option as this would increase instrument diameter and potentially give a higher prevalence of iPPROM.

Therefore, we propose in this work to rely solely on the intrinsic fetoscopic camera. We postulate that as the in-utero environment is dark and illuminated by a light source fixed with respect to the camera, a single fetoscopic image might contain enough information (a.o. in the form of the projected light cone) to estimate the orientation of the scene. The in-utero environment is different for each patient and acquiring a clear view is, sometimes, difficult. The surgeon may also change the scopes and the light intensity during an intervention, making it a challenging task to estimate placenta pose from these images. This work presents the use of convolutional neural networks (CNNs) to estimate the relative orientation of the placental surface with respect to the fetoscopic camera. Convolutional neural networks (CNNs) have found wide application in image processing due to their automatic feature extracting abilities. Via back-propagation convolutional filters in a multi-layer structure can efficiently learn specific features [13]. Olmschenk et.al. [17] demonstrated the use of CNNs to regress the pitch and roll of a camera using a single monocular image as the input. The architecture used in this work is achieved by progressively reducing depth of the first network presented in [17] to achieve fast inference time without reducing performance significantly. This architecture was used due to its simplicity and faster inference which can be crucial for our real time application. The regressed placenta pose is used to complete a feedback loop that realizes autonomous alignment of the flexible scope’s distal tip. This leads to a more flexible approach to ELA with improved usability as the surgeon does not need to bother how to deal with the distal tip.

## Methods

### Flexible actuated fetoscope for ELA

**Fig. 2:**
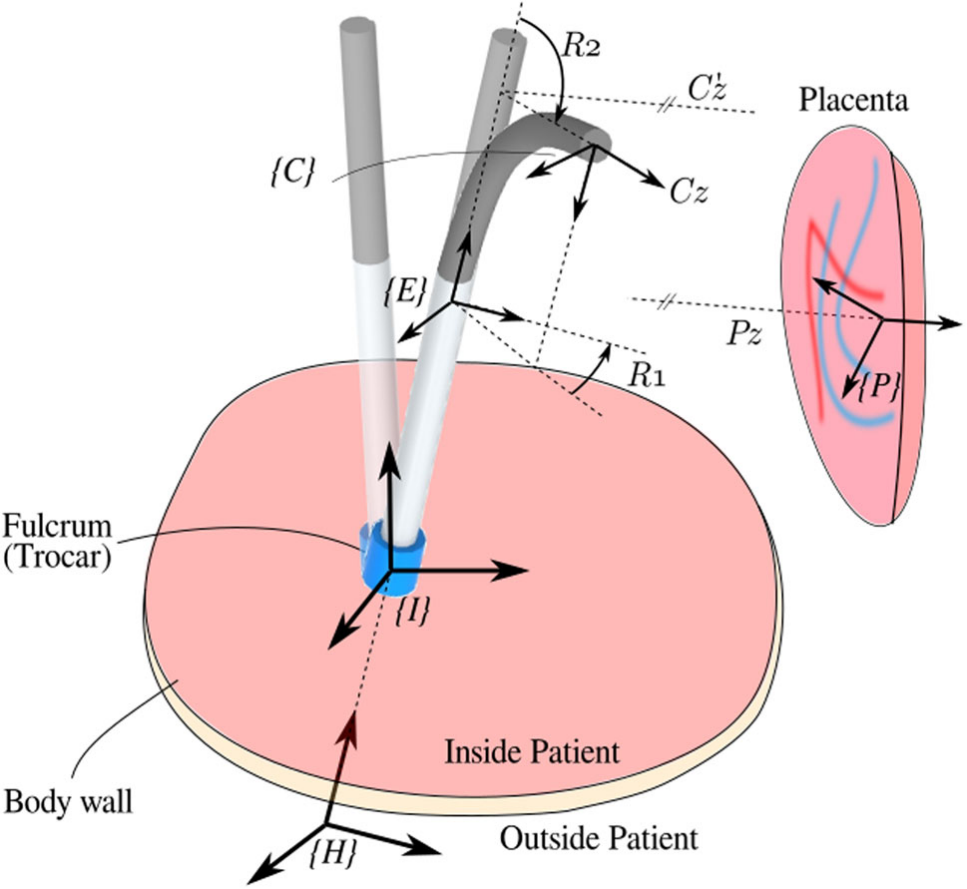
Sketch of intra-uterine cavity (upside down). Flexible instrument in straight, re-oriented and bent configuration. Automatic alignment of the distal flexible tip is achieved by aligning *z*-axes of camera frame $\{C\}$ and placenta frame $\{P\}$

ELA for TTTS is a minimally invasive procedure in which the instrument is introduced into the uterine cavity through an entry port or *cannula*. For effective ablation, the laser must be preferably oriented perpendicular to the anastomosing vessel and at a certain distance (typically 10–20 mm) to the vessel. This positioning task would require 6 DoFs, but, given that the laser beam is axi-symmetric and rotation of the fiber about this axis is redundant, only 5 DoFs are needed. However, the entry port acts as a fulcrum point that restricts the motion of the instrument by 2 DoFs. So from the total 6 only 4 DoFs are available for the surgeon to steer these instruments (from outside the patient’s body, Fig. 1). Figure 2 shows how introducing a 5th DoF in the form of a distal flexible tip suffices to reach all locations and orientations. Note that in the figure the instrument enters the body, from the bottom of the figure, into the uterine cavity (upper part). A frame of reference $\{I\}$ is attached to the entry port (fulcrum) and a frame $\{H\}$ to the instrument handle (that is manipulated by the surgeon). Frame $\{E\}$ is attached to the endpoint of the rigid portion of the instrument. Frame $\{C\}$ is attached to the camera, mounted at the instrument tip. The origin of $\{C\}$ coincides with the center of the 2D fetoscopic image. Frame $\{P\}$ is attached to the placental surface such that $-P_z$ is normal to the placental surface, which is locally represented as a plane. Vector $-P_z$ points inside the uterine cavity. The $\{P\}$ frame is placed arbitrarily on the $xy-plane$ of $\{P\}$ and coincides with the inner wall of the uterine cavity. The twist of $\{P\}$ about $P_z$ does not matter due to axi-symmetry. With 5 DOFs (in normal conditions), $C_z$ can be made parallel to vector $P_z$. A deviation of 5 around this orientation is deemed acceptable as it would not significantly affect the coagulation process and its efficiency. For rigid instruments that end at $\{E\}$ (and have only 4 DoFs), it is generally impossible to align $E_z$ sufficiently parallel to $P_z$. The figure shows that by introducing the extra bending DoF, perpendicularity can be aimed for.

**Fig. 3:**
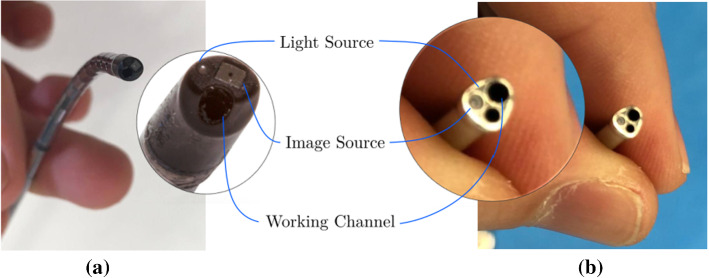
Distal tip of **a** in-house built fetoscope; **b** conventional ELA instrument

A functional instrument for ELA has 3 essential parts: an image source, a light source and a working channel for the laser fiber. Commercially available instruments come with a rod-lens or a fiber-bundle. Rod-lenses are used in straight rigid fetoscopes. Fiber-bundles are for curved semi-rigid fetoscopes [4]. Both imaging systems act as image conduits and are optically connected to an external camera. More recently chip-on-tip cameras appeared. These sensors are more flexible as only thin electrical cables need to be connected. Figure 3a shows an in-house-built instrument [1] that features an integrated NanEye camera [2] and a single optical fiber as light source. The bending of the flexible segment is controlled by actuating a McKibben Muscle [21]. Further, a motor is embedded in the handle. This motor rotates the, possibly bent, shaft about its longitudinal axis. With these 2 actuated DoFs the distal tip can be computer-controlled. Given a desired orientation (e.g., from a known pose of the placenta) a shared control approach becomes feasible, where the gross motion is controlled by the surgeon and the orientation of the distal tip is controlled by autonomously steering the 2 distal DoFs.

### Data generation

A large amount of labeled data would typically be needed to train a neural network to recognize the placenta pose. For in-utero applications, large datasets, leave alone large labeled datasets, are not available. Using dedicated sensors to capture the ground truth pose during actual clinical interventions is not feasible. Manual labeling is burdensome and impossible to perform accurately for pose estimation. To overcome these issues, we propose, similar to other works in the literature [7, 8, 22] to generate datasets for CNN training via a virtual reality simulator, and complement this with data from an in-silico model. To improve domain adaptation, a small set of roughly labeled samples from in vivo data is also included. This would introduce in vivo features in the trained network in a semi-unsupervised manner, while the accuracy is mainly driven by accurately labeled data. Images from all above data sources can be included, generating a rich dataset for training a neural network.

#### Data representation

Two actuated distal DoFs: *R*1, the rotation about the axis shaft and *R*2, the distal bending, are needed to adequately orient the distal tip. Given the relative short distance to the placenta and the small camera field of view (FoV) the placenta can be locally approximated to be planar. Recalling that the rotation about the axis $P_z$, normal to the plane, is redundant; therefore, only 2 DoFs are needed to describe the placenta inclination locally. The rotation matrix $^{C}\!R_P$

1 $$\begin{aligned} ^{C}\!R_{P} = \left[ ^{C}\!X_{P} \ ^{C}\!Y_{P} \ ^{C}\!Z_{P} \right] \end{aligned}$$

that represents the orientation of frame $\{P\}$ relative to $\{C\}$, is however a non-minimal representation. Other minimal representations, e.g., based on Euler angles, suffer from representational singularities. To avoid these problems, we propose a minimal representation based on the *x*- and *y*-components of vector $^{C}\!Z_{P}$:

2 $$\begin{aligned} \left[ x\_component, y\_component \right] = \left[ ^{C}\!Z_{P}.x , ^{C}\!Z_{P}.y \right] . \end{aligned}$$

**Fig. 4:**
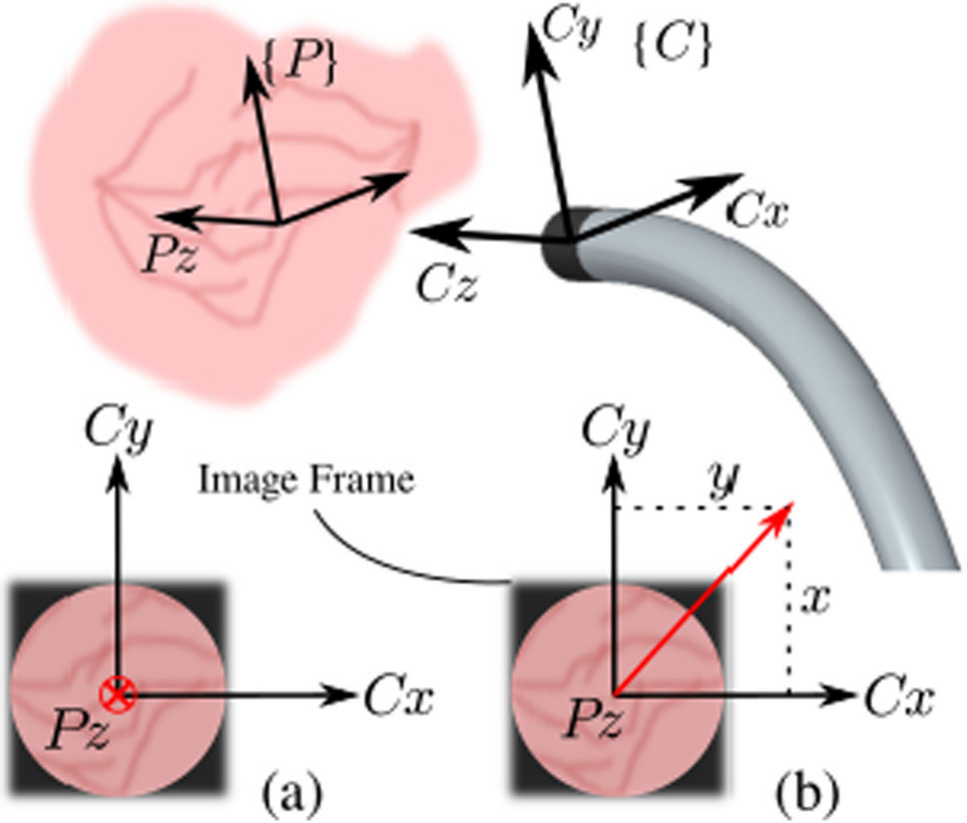
Representation of 2 DoFs for different image frames: camera **a** perpendicular to placental surface; **b** inclined w.r.t. placental surface

As shown in Fig. 4a, when the camera is perpendicular to the placenta, $P_z$ and $C_z$ are aligned and the projection of $P_z$ on the $C_{xy}$-plane is $\left[ 0, 0 \right]$. When the camera is not perpendicular (Fig. 4b) the projection of $P_z$ on $C_{xy}$-plane is a vector from the origin of $\{C\}$ to (*x*, *y*). This representation embeds sufficient information to re-orient the camera to become perpendicular to the placenta. It is also this information that will be used to label the data. Note that the two components simply follow from $^{C}\!R_P$ according to (1) and (2). Conversions to Euler angles, which could lead to multiple solutions or large jumps at zero-crossing, are hereby elegantly avoided. A further advantage is that this representation maps the DoFs within a Cartesian $[-1,1]\!\times \![-1,1]$ area, a feature that is beneficial for CNN applications as it simplifies regularization. Further, as shown in Fig. 2, when the axis of rotation of bending *R*2 aligns with $C_x$, the *x* and *y* components can be straightforwardly used as error signals for *R*2 and *R*1 in autonomous controllers.

**Fig. 5:**
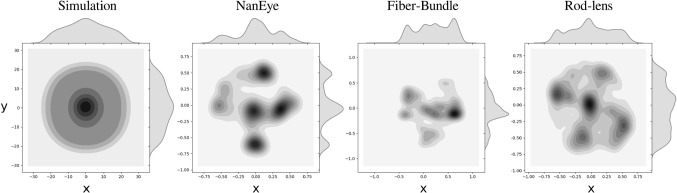
Distribution of training data in output space

**Fig. 6:**
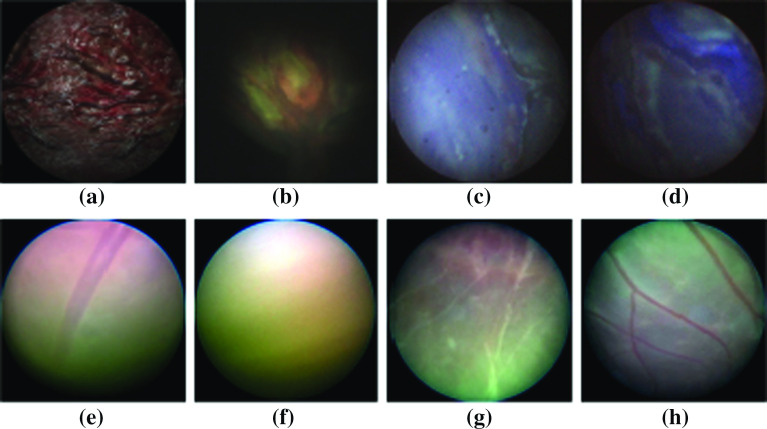
Samples from different image sources: **a** Simulation, **b** NanEye (in silico), **c** Fiber-bundle (in silico), **d** rod-lens (in silico), **e**–**h** in vivo from patients

#### Simulation dataset

Six real placenta high-resolution photographs were taken ex vivo from patients after delivery. These images were used as texture inside six virtual uterine cavities. A simulated camera and illumination were added to recreate fetoscopic views of the surgical scene. The relative position and orientation of the placental surface and the camera were programmatically altered creating various images with known ground-truth inclinations. *The Visualization Toolkit* (VTK), an open-source C++ toolkit, has been used to build up this simulation environment [18]. Note that thanks to the virtual reality it is possible to generate nice, uniformly distributed training datasets without too much human intervention. The distance between the camera and the placenta was varied between 10 *mm* to 50 *mm*, representative of the distances at which a surgeon typically operates. Meanwhile, the camera was rotated about the placenta in a conical trajectory such that $x\_component$ and $y\_component$ vary over a $-0.6$ to 0.6 range to cover the relevant space of output features (Fig. 5). In total 4 real placenta photographs were used to generate a training dataset containing 30,000 samples. 2 photographs were used to create a dataset of 14,000 samples, out of which, 1000 and 2000 samples were randomly selected for validation and test, respectively. A sample synthetic image from this dataset is shown in Fig. 6a.

#### NanEye phantom placenta dataset

**Fig. 7:**
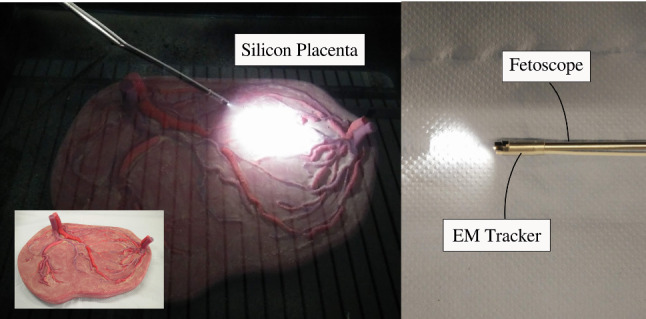
Silicon placenta phantom in water tank used to generate datasets with NanEye, rod-lens and fiber-bundle scopes

Images were captured from the NanEye camera that was integrated in the distal tip of the newly developed flexible scope [1]. An in silico model has been created by submerging a silicon placenta (Fig. 7) into a water tank. The tank was painted black and covered with an opaque lid producing an environment devoid of external light. The environment was then illuminated by a light source via the optical fiber embedded in the flexible scope. Overall, this closely mimicked the view during actual TTTS. As shown in Fig. 7, a 6-DoF electromagnetic tracking sensor (NDI, Waterloo [16]) is attached to the instrument tip. From the pose information and knowledge of the location of the in silico model, ground truth data were derived.

To train the network, enough variations in NanEye images and more specifically variations in relative orientations must be used. Figure 5 shows that for freehand motion the relevant space is sampled uniformly. To overcome poor sampling data-augmentation was applied by rotating the image, i.e., the origin of frame $\{C\}$, around its center, incrementally by 5. The corresponding values of $[x\_component, y\_component]$ were obtained by computing the rotated projection of $P_z$ along the image frame. A dataset of 11,000 samples was created out of which 500 and 1000 samples were taken for validation and test, respectively. A sample of such image can be seen in Fig. 6b. As NanEye being the image source from the proposed actuated flexible instrument, the model trained on this dataset will be used as the feedback sensor for the autonomous distal tip controller.

**Fig. 8:**
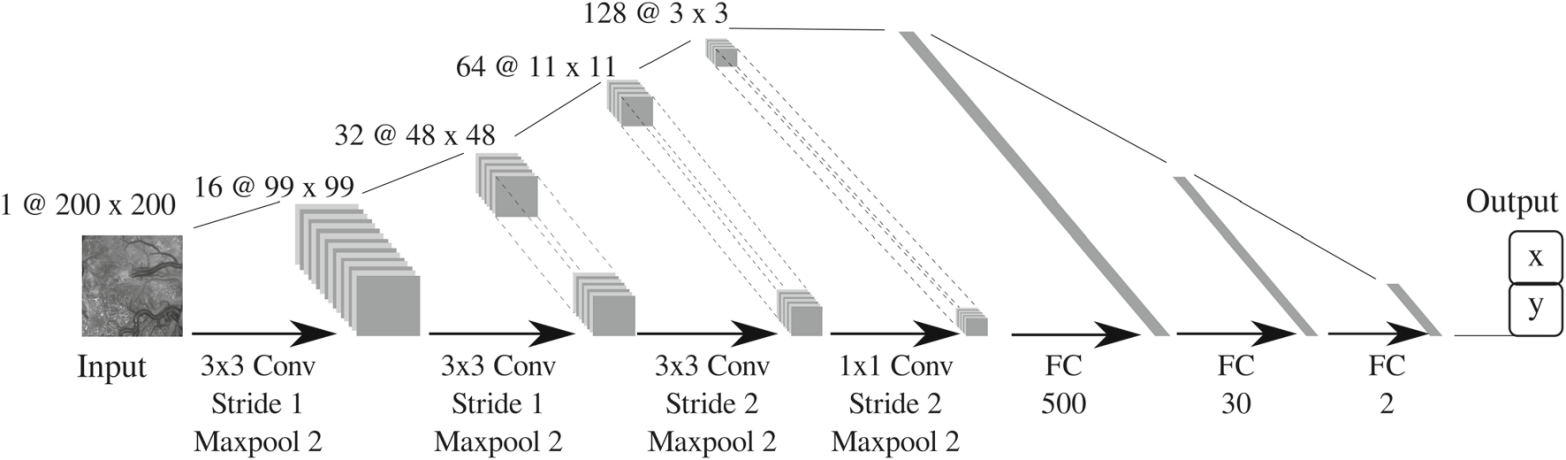
A schematic diagram of the employed CNN featuring 4 convolutional and 3 fully connected layers

#### Rod-lens and fiber-bundle phantom placenta datasets

Although a chip-on-tip camera is used in the present prototype, a fiber-bundle may be considered in the future. Figure 3b shows an instrument currently used in clinic. Typically, surgeons change between straight and curved scopes during an intervention. Hence, introducing image samples from a rod-lens scope and a fiber-bundle scope is also important. Two datasets were generated using a commercially available straight (rod-lens) and curved (fiber-bundle) scope. These datasets were generated and augmented using the same approach and phantom as in “NanEye Phantom Placenta Dataset” section. After merging, a single, henceforth referred to as RL-FB (rod-lens and fiber-bundle) dataset, was created. This dataset contains 23,400 samples. Sample images from the associated fiber-bundle and rod-lens scopes are visible in Fig. 6c, d.

#### Real data

Four fetoscopic videos of real interventions, representative for the viewing conditions when operating with a laser, were recorded in the operating room (OR). From these, 50 frames were hand-picked such as to represent: a wide range of relative inclinations and distances between the camera and the placental surface; natural noise in the images such as turbidity of the amniotic fluid, floating particles, and different zooming and lighting conditions. Four images can be seen in Fig. 6e–h. The ground truth for this data is unfortunately not available. The hypothesis is that, by including a small amount of these images during training, the network gets familiarized with real in vivo data examples and may hence perform better on such data, while the accuracy of inference is realized by the larger number of labeled examples. We do not expect that these in vivo images need too accurate labeling. Crude hand-labeling, e.g., by comparing the light pattern with the labeled images, is expected to be a pragmatic but acceptable way to move forward. Subsequently, each image frame was manually labeled by comparison with an image of known labels. The comparison was made based on the illumination pattern resemblance. This produces a dataset with samples sparsely situated in the output space. The distribution of the data was augmented using the same approach as in “NanEye Phantom Placenta Dataset” section, by rotating the images and calculating the corresponding values of the rotating $P_z$ projection. This dataset is henceforth referred to as the OR dataset. 40 images were used to create a training dataset (2880 samples), and 10 images were used to create the test dataset (720 samples).

### The network model

For this application, we propose a simple network, shown in Fig. 8, with 4 convolutional layers and 3 fully connected (FC) layers. The network architecture and hyperparameters were chosen based on inspiration from literature [17], and an iterative performance evaluation with the simulated training dataset. The performance on 3-channel color images was evaluated, but it was found that the network was able to learn also from a single channel grayscale image scaled down to $200\times 200$ pixels. This gave an advantage in the training and inference speed. By adopting a Stride 2 for maxpooling in layers 3 and 4, the filters were found to identify bigger features in earlier layers. As output layer, a FC layer of two units was used. As we are focusing on the regression of continuous output values, no softmax operation was applied to the last layer. Layers 3 to 6 have a dropout of $20\%$ for regularization. A Leaky ReLU activation function is used for all the layers because it was shown to be more effective for image processing applications than a normal ReLU [23] .

**Fig. 9:**
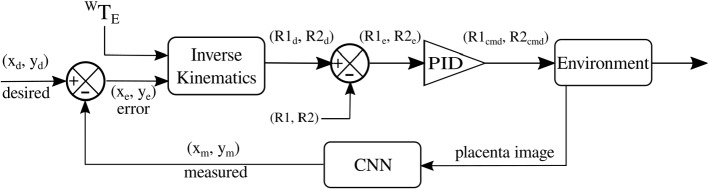
Shared control scheme for autonomous distal placenta alignment

### Shared control operation

The proposed shared control approach allows autonomous control of the flexible distal tip while having the gross motion under human control. Figure 9 shows the control scheme to steer the distal tip. The CNN is included in the return channel of the feedback controller. The CNN estimates the inclination from the fetoscopic images. The estimated parameters $(x_m,y_m)$ are compared to the desired values (for perpendicular inclination these are [0, 0]). Via the inverse kinematics block allows computing the desired joint values $(R1_d, R2_d)$ for given inclination errors $(x_e,y_e)$ and for a given pose $^{W}\!T_{E}$ of the instrument’s straight section. The joint errors are amplified via a PID to compute the steering commands $(R1_{\mathrm{cmd}}, R2_{\mathrm{cmd}})$ that are sent to the two actuators of the instrument. As the distal tip moves, placental images are updated. Then, the loop is closed again. Note that whenever the handle pose changes, $^{W}\!T_{E}$ changes and steering commands are updated.

**Fig. 10:**
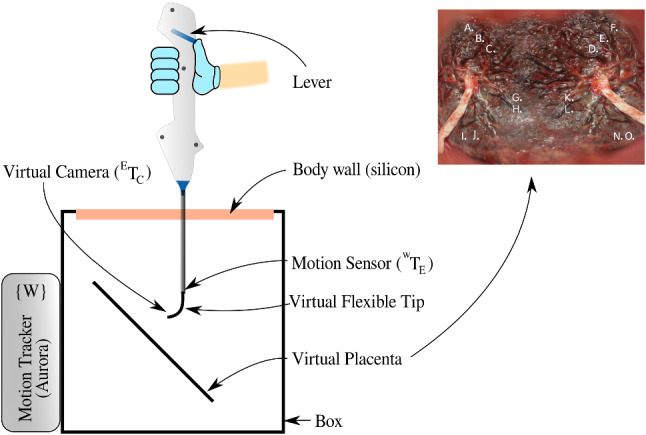
Experimental setup with motion tracking and mixed reality simulator. The placenta photograph with landmarks marked with letters

A single subject participated in the experiments with basic prior experience of the procedure in order to assess the difference of techniques without any bias.Three different configurations were tested. The task was executed with a normal straight scope, with a manually controllable bendable scope (using the lever) and with the above proposed shared control scheme. Each configuration was performed 5 times. The orientation of the scope relative to the ablation site was measured, and the deviation from perpendicularity was computed.

## Experiments

### Experimental setup

A mixed reality setup, shown in Fig. 10, is used for validating the shared control scheme [10]. The setup consists out of an endoscope, a box mimicking the body wall and a virtual reality simulator. The endoscope handle contains a lever that controls the bending of the flexible distal tip. This can be used for manual operation and is by-passed in shared control operation. A rigid straight metal tube with an electromagnetic (EM) sensor (NDI, Aurora) at the tip represents the instrument shaft. A magnetic field generator is placed behind an acrylic box. The motion tracking system captures $^{w}T_{E}$ in real-time and sends this to the virtual reality simulator at 40 Hz. Also the flexible segment is represented virtually. The position of the camera and light source at the flexible tip $^{E}T_{C}$ are computed from the lever pose and/or from the control command. The box mimics the uterine cavity. The upper surface, which is made out of mesh-infused silicon, mimics the body wall. The resistance that is felt corresponds closely to reality. The placenta, its inclination and the illumination are rendered virtually inside the box.

### Experimental protocol

An experimental task is designed to mimic the basic skills required for TTTS laser coagulation. The goal is to locate landmarks (marked by letters) on the surface of the placenta in a given sequence. For each landmark, the subject is asked to hold the laser target, visualized as a green dot in the center of this mark. He/she should maintain, in line with the clinical practice, the laser at an optimal distance of 10–20 mm from the surface. Then, the landmark is to be coagulated by pushing a foot pedal and triggering the laser. Figure 10 shows the placenta image that was used for the experiments.

## Results

The network was trained with a learning rate of 0.0001 and a batch size of 100. The Naneye dataset was trained separately due to reasons that the chip-on-tip image is square, while the RL-FB and in vivo datasets have a circular field of view. The simulation dataset served to design the model and for tuning the hyperparameters. This trained model was then used to initialize the weights for the other networks. The Adam optimizer was used for training [11]. From observing the performance on the validation during training, we found that the network stops learning after 50 epochs. On average, inference takes 4 ms with one GPU. Inference can thus be done in real-time at 250 Hz.

The accuracy is calculated by converting the predictions to angles. If both angles are within the acceptable range of $\pm \,5^\circ$, the predictions are deemed accurate. Table 1 summarizes the performance of the network on different training datasets. The third column shows the performance of the test dataset based on the corresponding training dataset, e.g., the test performance in simulation for a network trained on simulation data only (first row), the test performance on NanEye based on network trained on NanEye data (second row) and so on. In an ideal scenario, a single network should be robust enough to predict accurately on unseen data from any type of imaging source and in-utero conditions. The fourth column of Table 1 shows how these networks perform on the real in vivo test dataset.

**Table 1:** Performance of the network trained on different datasets

Training dataset	Epochs	Accuracy on test dataset (%)	Accuracy on in vivo test dataset (%)
Simulation	100	87	11
NanEye	80	81	NA
RL-FB	80	89	4
OR + RL-FB	80	85	60

Performance is measured on test datasets of the corresponding training datasets (column 3) and then on the in vivo test dataset (column 4)

**Fig. 11:**
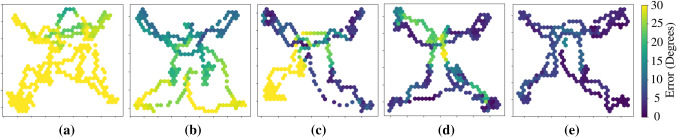
Error of laser firing angle over the placental surface. **a** Straight scope without flexible tip, **b** rigid scope curved at a permanent angle, **c** flexible tip with autonomous *R*2, manual *R*1, **d** flexible tip with manual control, **e** flexible tip with automatic control

**Table 2:** RMS and standard deviation of combined error from experiments in virtual reality simulator with straight rigid scope, flexible tip with manual control and flexible tip with autonomous control

Exp	Straight scope			Flexible tip-manual			Flexible tip-automatic		
	RMS Err (degrees)	Total RMS Err	Total STD Err	RMS Err (degrees)	Total RMS Err	Total STD Err	RMS Err (degrees)	Total RMS Err	Total STD Err
1	40.28	39.81	7.61	14.26	13.66	9.15	6.52	5.93	3.76
2	36.79			16.54			5.61		
3	43.22			13.98			7.34		
4	41.53			11.27			5.68		
5	37.23			12.25			4.50		

Figure 11 shows the heat map of the Combined error distributed over the surface of the placenta image. This error is

3 $$\begin{aligned} \mathrm{Combined \ error} = \arccos ( ^{W}\!Z_{C} \ \cdot \ ^{W}\!Z_{P} ), \end{aligned}$$

the angle between the normal vector to the placental surface $P_z$ and the vector $C_z$, without being dissolved into *x* and *y* components. The coordinates of the distal tip with respect to the virtual image plane were recorded along with the combined error. This Fig. 11 shows clearly which regions of the placental surface are more prone to perpendicularity errors during operation. In the laser ablation therapy, the surgeon not only aims to accurately ablate the target, but also navigates over the placental surface to locate more targets. The surgeon may also has to ablate in a line along the placental equator (Solomon’s technique [20]) which requires navigation while maintaining a perpendicular angle. Figure 11 shows these errors while ablation and navigation over the placenta surface.

## Discussion

Table 1 shows the performance of the network inferences. The network has 87% accuracy for the simulated dataset and 81% accuracy for the NanEye dataset. It means that 87% of the images are most likely to be within the acceptable range of prediction error in case of virtual imaging source. This is an acceptable rate of error for a real-time control running at 250 Hz (4 ms inference time). Similarly, for instruments employing NanEye image sensor, with proper filtering of inference data, real-time control can be achieved. It is worth noticing that the performance of the network on unseen in vivo samples increased from 4 to 60% by just adding a relatively small amount of samples in the training dataset. This will be very useful if it is decided, in future, to use fiber-bundle as an imaging source in shared-controlled flexible instruments. In future work, it would also be useful to add confidence measure of the model estimates as a parameter to be used for stable and reliable control.

Figure 11 shows some interesting results by showing the heatmap of combined error over the surface of the virtual placenta, giving precise locations of areas with a higher degree of error, which, in turn, are difficult to reach areas. Table 2 shows the RMS and the standard deviations of the combined error. Figure 4a is the case with a straight scope, without any flexible distal tip. The combined error for this configuration is reaching values of more than $30^\circ$ which is understandable given the placenta plane is placed at $45^\circ$ w.r.t to the entry point. Figure 4d is the error map for the manual operation of the instrument with the flexible distal tip. It is evident that the errors have significantly reduced by just adding the flexible tip at the same instrument for the same placenta orientation. From Table 2, a slight increase in standard deviation in the case of the manual flexible tip is observed. It may be inferred as an indication that this configuration is slightly more difficult for the user as compared to a rigid straight instrument. Figure 4e shows the best performing configuration in terms of combined errors in difficult to reach areas. This is due to the autonomous control of the distal flexible tip using the trained CNN for pose estimation. Table 2 shows very low values of standard deviation for automatic control indicating stability and also the RMS of combined error is significantly lower than the manual control of the same instrument.

## Conclusion

This work proposed a new shared-control approach for fetoscopic applications whereby a distal bendable tip is controlled autonomously via a CNN that estimates the relative orientation of the placental surface. The feasibility of placenta pose estimation through a CNN was demonstrated experimentally on a setup that mimics the real motion of an instrument or at least in a manner as it is expected to behave during the laser coagulation procedure. It was shown that autonomous distal tip alignment helped keeping the distal tip within an acceptable error range of $5^\circ$ for the designed tasks. The results with the autonomous controller were found to be superior to those of manual control. It was further found that adding a limited set of crudely labeled in vivo samples (OR dataset) to the training dataset (OR+RL-FB) improved the performance significantly compared to networks that were only trained on the RL-FB dataset. The instrument worked well within acceptable performance ranges. The instrument’s response was fast. The flexible tip adapted autonomously when operated in a shared control fashion.

Some errors were found at the center of the placental surface. It is believed that these errors are caused by the user moving away from the surface to obtain a larger overview upon the surface for better navigation. In principle these errors are not problematic as they typically do not take place during ablation. Note that this effect is reduced with the autonomous control. Latency of data acquisition and the poor quality of images in the instrument with chip-on-tip camera may cause challenges for real-time control. These challenges can be reduced by adding 2 optical fibers, instead of just one, for increased illumination. Also, using a fiber-bundle as image conduit for and external high resolution camera will improve quality of the acquired data.
